# Observations on the Use of Colchicum Autumnale in the Treatment of Gout, &c.

**Published:** 1826-01

**Authors:** 


*Observations on the Use of the Colchicum Autumnale in the Treatment of Gout; and on the proper Means of preventing the Recurrence of that Disorder.* By CHARLES SCUDAMORE, M.D. F.R.S., &c. Octavo, pp. 116. London, Longman and Co. 1825.

THE object of Dr. Scudamore is to present a clear outline of his opinions on the use of colchicum, and of the view which he takes of the theory and treatment of gout. He is convinced that this medicine, administered in its *mildest form*, and in conjunction with other remedies, is a valuable auxiliary in relieving the symptoms of that disease. Ample experience has taught him, that, if judiciously prescribed, it is innocent in its immediate effects, and innoxious to the constitution. He is aware that the colchicum has been much abused in its employment, and he fears that, unless its true merits be fairly represented, the sufferer will probably be deprived of one of the most important agents for the relief of gouty irritation. While he places a great value on the proper and combined use of colchicum for mitigating the symptoms of gout, he considers, that the treatment of the causes of this disease, is wholly to be found in other means. Such is his preface, stripped of its verbiage, and conveyed in the analytic style, which is preferred whenever it is practicable. Dr. Scudamore has already written well on gout, his experience of it must have been considerable, his opinions on the colchicum and the disease itself are entitled to attention, and as this vegetable production is now much in use, we shall transfer to these pages the substance of his present work.

Gouty persons have, for several years past, freely employed one or other preparation of colchicum, some have supposed that paralytic symptoms are an occasional consequence of its free exhibition, and hence the necessity of medical investigation to establish truth. The external appearance, denominated gout, is the effect of a cause existing in the system, but the true nature of this cause is a problem which eludes research. The fact is, however, indisputable, that certain individuals possess an hereditary disposition to gout, which is produced by exciting causes; whilst others acquire this disposition wholly from improper habits of living, acting upon peculiarity of constitution. We see people live irregularly, yet gout is not produced, but, on the general average, those who eat heartily of animal food, and drink freely of wine, spirits, or strong malt liquors, become subject to it, unless these causes be counteracted by active exercise, and a strength and equality of balance in the circulation, which prevent a congestion in the vessels of the viscera below the stomach. Gout, even in its first attack, is a compound disease; internal in its cause, external in its appearance. The paroxysm often seizes an apparently healthy subject in the night, and, if unmolested, may last for two or three months, injuring the constitution, crippling the whole frame, and exhausting the nervous system. This is a general description, and claims a licence against individual exceptions. The constitutional error which attends a first fit of the gout, is an overcharged state of the vessels of the abdominal viscera, and especially of the liver, which important organ is altered in its secretory action. The stomach deviates least, the appetite commonly continues natural, yet if sympathetic fever arise, thirst and anorexia follow; and ultimately, if improper habits be continued, dyspepsia will be produced. There is here too much abdominal corpulence; the secretions are changed; the evacuations are dark and fetid, and indicative of an irritating and acrimonious quality of the bile. That the conversion of chyle into blood is morbidly disturbed, is usually demonstrated by the condition of the urine, which possesses a higher specific gravity, is strongly animalized, and deposits dense sediments, consisting of the common salts in excess, and also animal matter. These appearances prove the process of assimilation to be imperfectly performed, and the blood neither natural nor healthy. Aperients and correctives, assisted by suitable diet, soon effect a cure in slight cases; and in some examples of a first fit, still simpler treatment succeeds—aperients, cooling diet, and rest. Where gout is acquired, care, temperance, and exercise have prevented its recurrence; but when the disposition is hereditary, such means will not entirely counteract a return, although of great collateral value.

"The gout, then, is a disease prone to return more or less frequently, to increase in severity, and to affect a great number of parts, as the constitution becomes more subjected to its decided influence.

"Even in its simplest form, as exemplified in a first fit, I have already stated that it is connected with that species of repletion which belongs to the vessels of the abdominal viscera, and chiefly of the liver: but, in its progress, it manifests this connexion much more strongly; and I do affirm that, in every long-established case of gout, the functions of the liver are more or less unhealthy; and, in a very large proportion of cases, the symptoms of gout are entirely supported by great derangement of the biliary system, and by an unhealthy condition of the intestinal canal. This disorder of functions is rendered manifest by the appearance of the excretions.

"The most remarkable cases of frequent relapse of gout which I have seen, have been those in which the error in question has been very conspicuous; and especially where such error has been increased and confirmed by improper treatment of the gout. This disorder is one which powerfully deranges the nervous system." 13.

The Greek physicians considered gout and rheumatism to be identical, and denominated the varieties of either according to situation:—*podagra* in the feet; *chiragra* in the hands; *pechyagra* in the elbow; *gonagra* in the knee; *dentagra* in the teeth; *cleisagra* in the articulations of the clavicles; *omagra* in the articulations of the humerus; *rachisagra* in the spine; and *tenontagra* in the large tendons.* The Romans adopted these terms; and it is obvious these opposite diseases were treated alike by the ancients, according to the doctrines of the humoral pathology, and appearance of acute and chronic symptoms. Hippocrates advised diluents and purgatives, and cold water to the inflamed part to induce numbness, and relieve pain. The ancient physicians prescribed for gout and rheumatism with boldness, and often called in the aid of venesection and purgation. It was a favourite practice to use cold applications to parts too warm, and warm to those which were too cold. Alexander Trallian theoretically divided gout, agreeably to the four humours of the body, into the bilious, sanguineous, pituitous, and melancholic, and his remedies, blood-letting, cathartics, aloes, scammony, seeds of wild cucumber, and hermodactyls, were adapted accordingly. Thus it is clear, from these and other sources, that this disease was actively treated even in the dawn of medical science. Sydenham wrote on gout in 1683, and to him we owe a correct description of it, but his theory and practice, founded on the humoral pathology, were unavoidably defective. The application of cold water to gouty inflammation, warmly eulogised by Dr. Kinglake of Taunton, has now scarcely any advocates, except under certain limitations. The eau medicinale came into use in this country fifteen years ago, its undeserved fame soon declined, and has now wholly passed away. The colchicum autumnale was the next remedy offered to public attention, and it may be considered as tantamount to the hermodactyl of the ancients.

"I had made some private trials of this medicine in gout, for a short period before I met with the account given of its effects by Mr. Want, in the Medical and Physical Journal, No. 185.

"From comparative trials with the powder of the root; the tincture; and the acetic preparation, I soon became convinced of the remarkable

* Cœlius Aurelianus, lib. v. cap. 2.

mildness of the latter, and the preference due to it in that particular. By joining it with magnesia and a neutral salt, such as the sulphate of magnesia, I found a combination inoffensive to the stomach, and remarkably certain in its operation on the bowels. I was at a very early period impressed with the conviction, that the most favourable use of the colchicum consisted in making a selection of this the mildest preparation, and of employing it only in combination with other medicines of an aperient and corrective nature. It was my object to borrow, in a safe manner, from the power which this medicine might exert over the symptoms of the disorder, but not to regard it as a lasting agent of cure.

"During my progressive experience in the treatment of gout, the asserted power of colchicum in curing the disorder became more and more promulgated. Sir Everard Home introduced into use a vinous infusion, which he directed to be made by macerating two pounds of the fresh roots with twenty-four ounces of sherry wine, in a gentle heat for six days, the spirit being previously carried off by heat.* He considered this medicine to be identical with the eau medicinale, and that each remedy acted as a specific cure for the gout. It appeared to him, that when this infusion was administered in its transparent state, after the removal of the deposit which it gradually makes, it acted with much greater mildness; and he extolled it as a complete and successful remedy." 24.

In the year 1815, Dr. Wilson† recommended his tincture for gout, and expressed himself to be dissatisfied with the effects of colchicum. To this Reynolds' specific followed. The eau medicinale is most active; next, Wilson's tincture; and, lastly, the specific of Reynolds; and, in the opinion of our intelligent author, who has performed numerous experiments, which cannot here be detailed, they all are preparations of colchicum. Three drachms of eau medicinale, and six of Wilson's tincture, each given in two doses, proved fatal to dogs; and all the strong preparations of colchicum had a similar effect.‡ It is indisputable that every one of them is a most powerful agent, and demands a cautious and moderate administration:—they may, and do, subdue the apparent disease in a large dose, but they act violently on the stomach and bowels, procure a full and immediate influence on the nervous system, and lay the foundation for severe and oft repeated attacks, which either embitter or endanger life.

---

"* This watery rather than vinous liquor proved so unfavourable a menstruum, that it has not been employed."

† See Wilson on Gout, First Edition, p. 42.

‡ The acetic preparation of colchicum would seem to be exceedingly mild in its operation, since twenty-four drachms, mixed with magnesia, were given in two doses to a dog without occasioning illness, and only moderately acting on the bowels and kidneys.—*Rev.*

"The *acetum colchici*," then, is the favourite medicine with Dr. Scudamore, and he entertains a high opinion of sarsaparilla, and of the sulphate of quinine as tonics. He considers it to be an axiom of importance that mercury should never produce mercurial fever in gouty persons; for the object from it is immediate purgative action, or an alterative one, in conjunction with aperients. When the alvine excretions are dark and fetid, and the urine, on cooling, deposits pink or lateritious sediments, he prefers a moderate dose of calomel, with a grain of James's powder, some compound extract of colocynth, and three or four grains of extract of poppy, which are to be taken at bed time, and followed by a suitable aperient in the morning. This method having been continued, with occasional intermissions, until the secretions are no longer vitiated in appearance, it will be expedient to employ the alterative form of mercury, *e. g.* the *pilula hydrargyri, hydrargyri oxydum cinereum*, or the *pil. hydr. submur. comp.* conjoined with the *extr. colocynth. comp.* or *extr. rhei.* and succeeded in the morning, regularly, by a gentle aperient, which should neither weaken the stomach nor excite nausea. The *pilula hydrargyri* is the mildest of these mercurial preparations, and half a grain of ipecacuan, and a grain or two of Castile soap form, on many occasions, a useful addition. Ipecacuan is preferable if it be wished to increase secretion from the intestines; the powder of squills, if from the kidneys; and James's powder, if to promote a free cuticular action. The compound aloetic decoction, with camphor mixture, is favourable to the stomach, as a morning aperient, when a cordial property is desired; and, when a cooling quality is sought, the sulphate of magnesia *in mistura camphoræ*, with *sp. ætheris nitric.*; or an infusion of senna, with manna and salts; or the salts alone. To improve the tone of the stomach, sarsaparilla, or the sulphate of quinine is to be prescribed during the middle of the day. Such are the general principles, upon which the author treats gout, subject to necessary changes in particular individuals;—for, let it not be forgotten, whether in a first attack, or where the constitution has become habituated, and suffered greatly, he affirms the disease to be "more or less connected with, and depending upon, a wrong action of the liver, and a faulty condition of the bowels; and that this state of disorder, and the gout, stand in the relation of cause and effect."

"When the biliary functions are completely restored to health, the mercurial alterative should either be laid aside, or used only occasionally; but when the action of the liver and of the intestinal canal is permanently in error, as I have pointed out, the value of mild doses of mercurial medicine, in conjunction with aperients, is, in my mind unquestionable. The alterative administered at bed time, and the aperient before rising in the morning, afford real benefit and sensible relief. In this manner also, an increased action of the bowels may be maintained for a long time without producing debility; because the morning medicine does not rob the patient of nutriment, while it ensures the removal of vitiated accumulation from the bowels. Aperients given during the day, if long continued, tend to reduce the strength and flesh; by preventing a sufficient stay of the food for perfect digestion." 93

In gouty persons, and others apparently not so, examples of constitutional indisposition manifest themselves, but which are so obscurely marked, that, unless the nature of the excretions from the alimentary canal be attentively and regularly investigated, no true knowledge of such indisposition can be obtained. Chronic cases of this kind are very common. The patient can scarcely describe his complaint; his bowels are often regular; he speaks of nervous depression and occasional lassitude; the sleep is unrefreshing, and he probably suffers slight pain or uneasiness in the hypochondriac region, shoulder, or scapula. The appetite is moderately good, yet digestion is imperfect. Invariably a loss of flesh may be detected, and the natural firmness in the muscles of the limbs is partly wanting. Here the alvine and urinary excretions must be inspected daily, and the united influence of regimen and medicine should be persisted in until the restoration of healthy functions and of general personal appearance. The proper treatment has been already detailed.

The prophylaxis of gout now claims notice. Although this disease will sometimes occur in defiance of all human endeavours, yet none will deny that it is, in a great degree, the offspring of indulgence and irregularity. Diet is a most important consideration. Materials tending to acetous fermentation, and to excessive nutrition, ought to be equally interdicted. A dinner of turtle might be digested with ease, but it would contribute to the formation of too much blood. Even if the appetite be good, moderation in eating should be practised, which will guard against a plethora that may introduce a gouty paroxysm. Dyspeptic symptoms are common and evident, and need appropriate means. Salmon and stewed fish are objectionable; also pork; all meat which is not tender; particularly hard salted meats, pickles, and rich pastry, with confectionary. Plain puddings or fruit ones; semolina, rice, or bread; oysters, when in season, and the beard part removed; and simple well made gravy soups, are admissible, with due restrictions. Sherry, Madeira, or Port, a little diluted with water, are to be recommended. The aid of medicine is valuable as a preventive of gout. Repletion of the vessels of the abdominal viscera ought to be guarded against, and of this the sufferer can judge, by watching the size of the whole abdomen, inasmuch as every protracted fit is preceded by notable signs of fulness, and an increase of bulk, and occasionally by some warning feelings of general oppression of the system, arising from an over-charged state of the circulation. Gouty persons, therefore, should, however regular their bowels may be, take an aperient at least once a week throughout the year, and if corpulent, or of a full habit, twice in the seven days. Sometimes the daily use of a dinner-pill answers very well, *e. g.* five or six grains of the *pilul. rhei compos. (Pharmac. Edin.)* For the majority of patients disposed to gout, the occasional exhibition of more efficient aperients will be desirable and beneficial; as, for instance, an electuary, composed of *pulv. scammon. comp.*, *potass. supertartrat. et confect. sennæ,* with syrup of orange-peel; or pills of scammony, colocynth, and soap. The *pilula hydrargyri* may now and then be given in combination.

When, as if mocking all prophylactic endeavours, a severe paroxysm of gout occurs, it is to be treated with a combined and comprehensive plan of medicines, applicable to the symptoms of suffering and disorder. The various nostrums, or the strong preparations of colchicum, are to be neglected. The dose of *acetum colchici* is from half a drachm to a drachm and a half, and very commonly a drachm, and when neutralized and joined with an aperient salt, it generally agrees with the stomach, and never produces constitutional nervousness or other uneasiness; but when the bowels are irritable the salt may be omitted. A pill, made with the extract obtained from the *acetum colchici* by inspissation, may be occasionally and advantageously substituted. One grain of this extract is equivalent to eighty minims of the acetum. Moderate doses of calomel, with James's powder, and the compound extract of colocynth, form the most efficient aid to the colchicum draught, or pill for the evacuation of vitiated secretions. For pain at night opium should be prescribed, and, to induce free cuticular action, James's powder or tartarised antimony may be added. Where a certain degree of depression and debility occurs in constitutions remarkable for the nervous temperament in consequence of the sufferings produced by the paroxysm, and even from the influence of the necessary treatment, restorative medicines and a nutritious diet, with the occasional use of correctives and aperients, are indispensable, even prior to the cessation of acute symptoms. Again, in all cases where the stomach is impaired in its nervous energy, or the system is relaxed, restorative means are to be employed. For which purposes sarsaparilla and quinine are admirable agents. Local treatment is highly important, not only immediately, but subsequently. Gouty inflammation, unchecked, occasions organic mischief in the affected textures. The evaporating lotion is to be applied, tepid, by linen rags, or as a poultice, mixed with grated bread, which commonly alleviates the burning sensations, shortens the duration of the inflammation without repelling it, and prevents the depositions arising from morbid secretion, and the ultimate thickening of the locomotive textures. Immediately after the subtraction of preternatural heat, the part is to be sponged for a few minutes with the lotion several times daily; and if required, for the removal of tenderness, a poultice may be ordered at night. The stiffness and weakness of the limbs, consequent to repeated attacks, demand a particular treatment, such as the different kinds of baths, according to the nature of the lameness; sponging with tepid salt water; friction, with liniments; bandages; and a systematic mode of hand-rubbing and shampooing. The latter are imperiously called for in permanent weakness and lameness, and, in union with auxiliaries, they afford relief, or cure, under circumstances the most unpromising.*

"I cannot refrain from again expressing my opinion, that there is not one important disease which admits of so much certain relief from medical treatment, as the gout.

"If proper attention be given when the disorder first invades the constitution, lameness, and other distressing results of neglect, may certainly be prevented.

"The returns of the complaint being induced by many remote causes, great care and watchfulness are required on the part of the patient. When the constitution has become completely gouty; and more especially, when the tendency to relapse has been increased by the influence of empirical treatment; very prompt success cannot reasonably be expected from the use of regular medicines.

"But in the worst cases I do affirm, that solid and lasting benefit will be derived from instituting regular principles of practice, varied in the adaptation of remedies according to the difference of constitution, the nature and force of the symptoms, and the attendant collateral circumstances of each individual case." 116.

We have now completed a full and faithful analysis of this

* In addition to the benefit afforded to the limbs, observes Dr Scudamore in a note, the influence of various kinds of baths upon the constitution is highly important. The vapour bath is a very efficacious agent in changing the functions of the skin; and it often, also, materially improves the action of the kidneys and of the bowels.

work, without the interposition of our own sentiments or the indulgence of criticism: and it is our duty to express a favourable opinion of it:—the facts are valuable, the doctrines sound, the practice excellent, and the directions judicious, but the style is loose, feeble, and incorrect, injured by want of arrangement, and disgraced by frequent repetitions.

